# Vangl2 disruption alters the biomechanics of late spinal neurulation leading to spina bifida in mouse embryos

**DOI:** 10.1242/dmm.032219

**Published:** 2018-03-01

**Authors:** Gabriel L. Galea, Oleksandr Nychyk, Matteo A. Mole, Dale Moulding, Dawn Savery, Evanthia Nikolopoulou, Deborah J. Henderson, Nicholas D. E. Greene, Andrew J. Copp

**Affiliations:** 1 Developmental Biology of Birth Defects, UCL GOS Institute of Child Health, London, WC1N 1EH, UK; 2 Cardiovascular Research Centre, Institute of Genetic Medicine, Newcastle University, Central Parkway, Newcastle upon Tyne, NE1 3BZ, UK

**Keywords:** Neural tube, Vangl2, Biomechanics, F-actin, Mouse, Embryo

## Abstract

Human mutations in the planar cell polarity component VANGL2 are associated with the neural tube defect spina bifida. Homozygous Vangl2 mutation in mice prevents initiation of neural tube closure, precluding analysis of its subsequent roles in neurulation. Spinal neurulation involves rostral-to-caudal ‘zippering’ until completion of closure is imminent, when a caudal-to-rostral closure point, ‘Closure 5’, arises at the caudal-most extremity of the posterior neuropore (PNP). Here, we used *Grhl3^Cre^* to delete Vangl2 in the surface ectoderm (SE) throughout neurulation and in an increasing proportion of PNP neuroepithelial cells at late neurulation stages. This deletion impaired PNP closure after the ∼25-somite stage and resulted in caudal spina bifida in 67% of *Grhl3^Cre/+^Vangl2^Fl/Fl^* embryos. In the dorsal SE, Vangl2 deletion diminished rostrocaudal cell body orientation, but not directional polarisation of cell divisions. In the PNP, Vangl2 disruption diminished mediolateral polarisation of apical neuroepithelial F-actin profiles and resulted in eversion of the caudal PNP. This eversion prevented elevation of the caudal PNP neural folds, which in control embryos is associated with formation of Closure 5 around the 25-somite stage. Closure 5 formation in control embryos is associated with a reduction in mechanical stress withstood at the main zippering point, as inferred from the magnitude of neural fold separation following zippering point laser ablation. This stress accommodation did not happen in Vangl2-disrupted embryos. Thus, disruption of Vangl2-dependent planar-polarised processes in the PNP neuroepithelium and SE preclude zippering point biomechanical accommodation associated with Closure 5 formation at the completion of PNP closure.

## Abstract (precis)

**Summary:** Disruption of Vangl2-dependent planar-polarised processes in the posterior neuropore (PNP) neuroepithelium and surface ectoderm preclude zippering point biomechanical accommodation associated with Closure 5 formation at the completion of PNP closure.

## INTRODUCTION

Neural tube defects (NTDs) are severe neurodevelopmental disorders which affect approximately one in every 1000 births (Morris et al., 2016). NTDs arise owing to failure of NT closure in early gestation. In mammals, NT closure initiates at multiple sites referred to as ‘closure points’, with Closure 1 at the hindbrain/cervical boundary initiating cephalic and spinal neurulation. Spinal NT formation involves a wave of ‘zippering’ that moves in a rostral-to-caudal direction along the elongating spinal axis (Nikolopoulou et al., 2017). The region of closing NT caudal to the ‘zipper’ is called the posterior neuropore (PNP). It is composed of elevating lateral neural folds that flank a midline neural plate. During spinal neurulation, the neural folds become apposed medially, narrowing the PNP and uniting at the zippering point to create the roof of the newly formed NT, which is covered by surface ectoderm (SE). Failure of this closure process leads to open spina bifida (myelomeningocele).

Closure progresses along much of the length of the future spinal cord through the combination of neural fold medial apposition and zippering, with narrowing and shortening of the PNP. When completion of PNP closure becomes imminent, a *de novo* closure point, referred to as ‘Closure 5’, forms at the caudal extremity of the embryo (Galea et al., 2017). This recently described closure point is characterised by a change in PNP shape from a spade-like to elliptical morphology, in which the elevated neural folds are encircled by an F-actin ring-like structure with cytoskeleton-rich protrusions forming at the caudal canthus of the PNP. Such protrusions have been found to characterise the main zippering point (Rolo et al., 2016), and their presence at Closure 5 suggests that it also forms a (caudal-to-rostral) zipper. Closure 5 biomechanically contributes to neural fold apposition as its laser ablation causes rapid widening of the PNP (Galea et al., 2017). However, the mechanisms underlying Closure 5 formation and its roles in the completion of spinal closure are largely unknown.

The mechanisms underlying initiation of spinal closure are crucially dependent on planar cell polarity (PCP)/van Gogh-like (Vangl) 2 signalling. Global deletion of Vangl2 precludes convergent extension movements required to narrow the neural plate and form Closure 1 at the start of neurulation (Ybot-Gonzalez et al., 2007b). Consequently, *Vangl2^−/−^* embryos develop fully penetrant craniorachischisis (Ramsbottom et al., 2014), precluding analysis of Vangl2 roles in spinal neurulation. Nonetheless, various lines of evidence suggest that Vangl2 does play substantial roles in spinal neurulation, subsequent to closure initiation. In humans, unique Vangl2 mutations have been associated with cases of lumbosacral NTDs (Kibar et al., 2011). In mice, heterozygous dominant-negative loop tail (*Lp*) mutation of Vangl2 causes distal spina bifida in a large proportion of embryos when combined with homozygous deletion of *Sdc4* (Escobedo et al., 2013), or heterozygous deletion of *Ptk7* (Lu et al., 2004) or *Sec24b* (Merte et al., 2010). Given that the locations of spina bifida lesions reflect the somite level at which PNP closure ceases, each of these examples of distal spina bifida suggest that Vangl2 is involved in late spinal neurulation, although its roles in completion of PNP closure are poorly understood.

The cellular functions of Vangl2 in neurulation have predominantly been studied in lower vertebrates and during early stages of mouse neurulation. In the zebrafish neuroepithelium, Vangl2 directs anterior-posterior cell polarisation and coordinates the direction of cell division (Ciruna et al., 2006). A well-established role of PCP signalling in various models is its regulation of the actin cytoskeleton, at least in part by recruiting Rac GTPases to adherens junctions (Lindqvist et al., 2010). Cytoskeletal regulation by Vangl2 is likely to be of relevance to spinal closure, given that combined haploinsufficiency of Vangl2 and the actin regulator Shroom3 also causes distal spina bifida in mice (McGreevy et al., 2015). This is consistent with the evolutionarily conserved role of PCP signalling in directing formation of supracellular F-actin cable-like structures extending across neighbouring cells (Ossipova et al., 2015b; Monier et al., 2010). Apical F-actin cables which form through a PCP-dependent mechanism have been shown to contribute to bending of the chick neural plate (Nishimura et al., 2012). F-actin is normally enriched in the apical domain of the mouse neuroepithelium, and supracellular F-actin enrichments have been described in the anterior neural folds at early stages of mouse neurulation (McGreevy et al., 2015). These apical enrichments are disrupted by the *Lp* mutation (McGreevy et al., 2015). Consistent with this disruption of apical F-actin, neuroepithelial cells in *Lp* homozygous mutant mouse embryos are deficient in the process of apical neighbour exchange involved in convergent extension movements (Williams et al., 2014). Similar roles of Vangl2 in cellular partner exchange have also previously been documented in lower vertebrates, such as in epithelial radial intercalation in *Xenopus* (Prager et al., 2017; Ossipova et al., 2015a)*.*

Many of these studies in mice have depended on use of the *Lp* allele, which has proven to be a tremendously valuable genetic tool but is limited by its constitutive, global nature. The development of a floxed allele of Vangl2 (*Vangl2^Fl^*) permits Cre-driven targeted deletion (Ramsbottom et al., 2014). In this study, we selectively deleted Vangl2 using the previously reported (Camerer et al., 2010) *Grhl3^Cre^*. Expression of this Cre driver follows that of the endogenous *Grhl3* gene, with early expression in the SE from embryonic day (E) 8.5 (when Closure 1 occurs) later followed by scattered expression in the neuroepithelium from E9 (Camerer et al., 2010; Rolo et al., 2016). Thus, in this study we disrupted PCP/Vangl2 signalling in the *Grhl3* expression domain in order to interrogate this pathway's roles in spinal neurulation.

## RESULTS

### The neuroepithelium and surface ectoderm of mid-neurulation mouse embryos display features of planar polarity

**Fig. 1.**
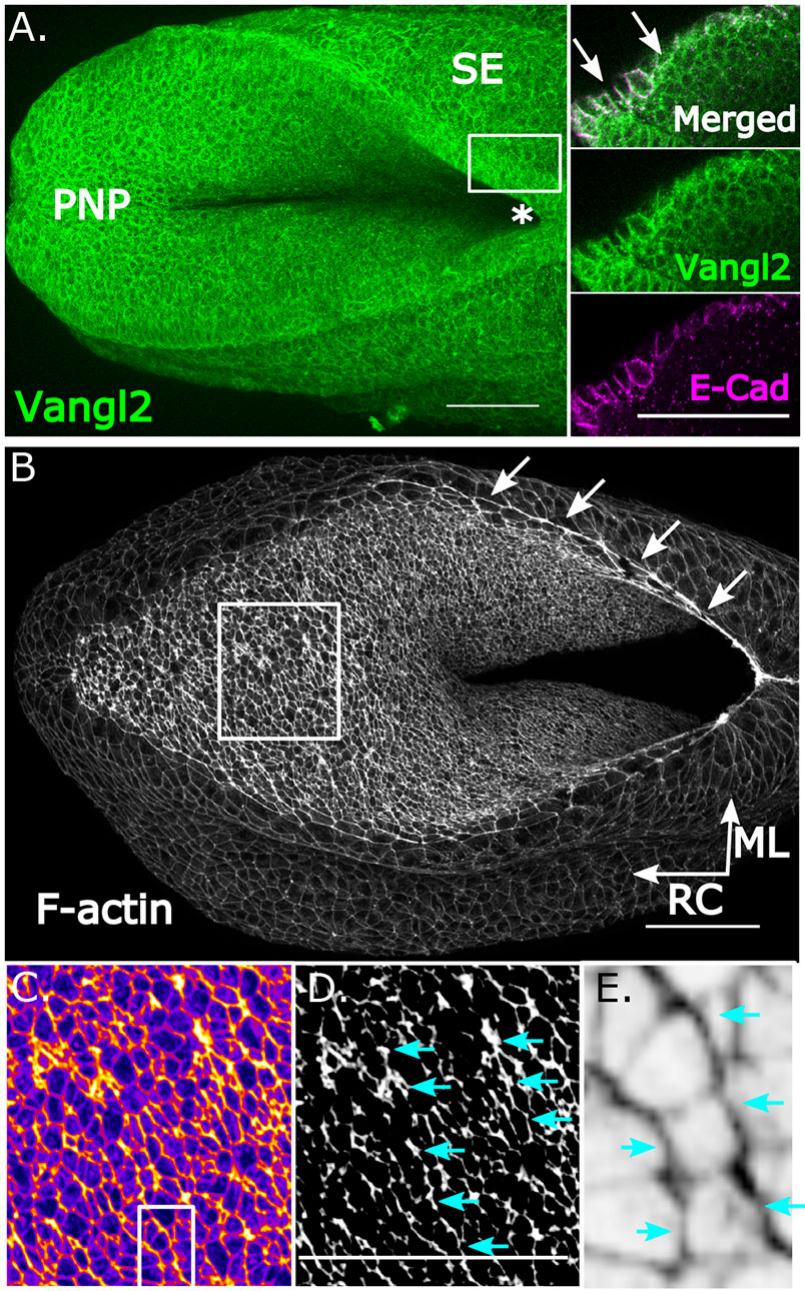
**The neuroepithelium of mid-neurulation mammalian embryos displays planar-polarised F-actin profiles.** (A) Whole-mount immunofluorescence image of an E9.5 mouse embryo stained for Vangl2, showing expression in the posterior neuropore (PNP) as well as surrounding surface ectoderm (SE). Vangl2 colocalisation with the SE marker E-cadherin (E-Cad) is shown in an optical slice (located within the white line box next to the zippering point, which is indicated by an asterisk). (B) Representative phalloidin-stained E9.5 mouse PNP showing the presence of a supracellular F-actin cable along the neural folds (arrows) and F-actin enrichment on the apical surface of the PNP (white line box, enlarged in C-E). (C) Fire LUT of the boxed area in B, highlighting F-actin enrichment in red. (D) The same image shown binarised to illustrate ML profiles (cyan arrows) of enriched borders. (E) Enlarged region from the boxed area in C, showing F-actin enrichment of ML-oriented cell borders (cyan arrows) relative to RC-oriented borders between them, forming a ladder-like pattern. Scale bars: 100 µm.

The PNP of mid-neurulation mouse embryos shows features of PCP in both the neuroepithelium and SE. Both these cell types (as well as the intervening mesoderm) express Vangl2 (Fig. 1A). We have recently identified a supracellular F-actin cable (Fig. 1B) running along the borders of the neural folds, where it colocalises with the SE marker E-cadherin (Galea et al., 2017). This cable is also enriched in phosphomyosin light chain (pMLC) II (Fig. S1). Examination of apical F-actin in the PNP neuroepithelium revealed a second type of F-actin enrichment: mediolaterally oriented multicellular cell border enrichments (Fig. 1B-E). Similar actomyosin enrichments were previously identified in the mouse cranial neuroepithelium at earlier developmental stages (McGreevy et al., 2015) and in epithelia of embryos from other species (Nishimura et al., 2012), and have been described as F-actin ‘cables’. In order to distinguish these neuroepithelial apical F-actin enrichments from the much more extensive cable that can extend over 0.5 mm along the neural folds of the mouse PNP, we will refer to the mediolateral arrangements as ‘apical profiles’. pMLCII staining in the apical neuroepithelium is more heterogeneous than that of F-actin, but similarly forms mediolateral profile-like enrichments (Fig. S1).

**Fig. 2.**
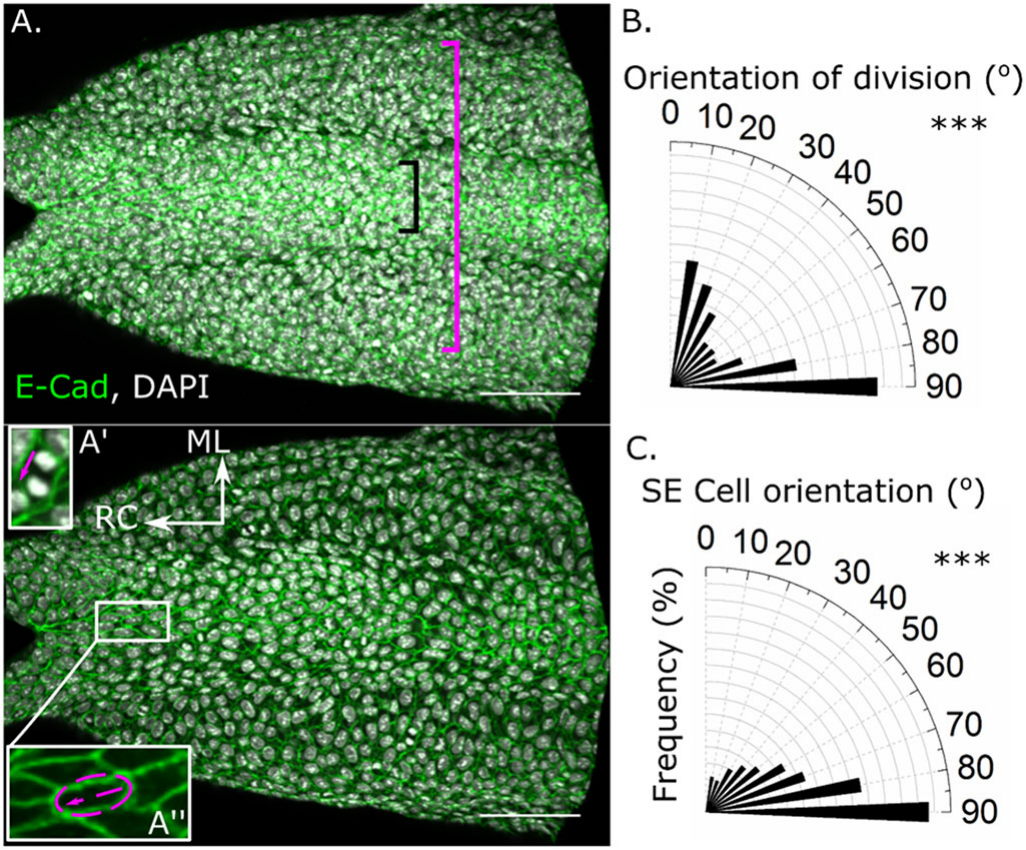
**The surface ectoderm of mid-neurulation mouse embryos displays directionally polarised cell divisions and cell bodies.** (A) Representative dorsal view of an E9.5 mouse embryo whole mount stained for the surface ectoderm marker E-cadherin (E-Cad). The same image is shown before (top) and after (below) surface subtraction segmentation using an in-house macro, allowing selective analysis of the tissue surface (e.g. exclusion of DAPI signal from deeper cells). Although the cell dimension and orientation analyses could only be performed in the relatively flat region of the dorsal midline (black bracket), the angle of division analyses were performed over a larger region (magenta bracket), owing to the small number of divisions directly in the flat region, independently replicating a previous report (Sausedo et al., 1997). (A′) Representative mediolateral (ML) division (RC, rostrocaudal). The magenta arrow indicates the orientation of division. (A″) Illustration of the determination of cell orientation based on the long axis of an ellipse around the cell borders. (B) SE divisions in the dorsal SE are preferentially RC and ML oriented. Data represent 101 divisions from eleven 13- to 17-somite-stage embryos. (C) The long axes of SE cells on the embryonic dorsal midline rostral to the zippering point are polarised in a predominantly RC orientation. Data represent 876 cell-long axes within one field of view rostral to the zippering point from five 15- to 17-somite-stage embryos. ***P<0.001 versus the expected proportions in a random distribution, 90° is RC. Scale bars: 100 µm.

Analysis of putatively planar-polarised properties in the SE is substantially confounded by deeper tissues, which are also visualised by confocal micrographs (Fig. 2A; Fig. S2A). To circumvent this, we developed an in-house macro, implementable in ImageJ, which allows selective analysis of the SE as the top cell layer (Fig. 2A). Using this image processing tool, we initially analysed the orientation of SE divisions, previously reported to be preferentially mediolaterally (ML) and rostrocaudally (RC) polarised (Sausedo et al., 1997). Preferential ML and RC orientation of SE cell divisions occurring rostral to the zippering point was confirmed in these analyses (Fig. 2B). The analyses also revealed that SE cells overlying the closed NT along the embryonic dorsal midline are preferentially RC oriented (Fig. 2A,C); however, these analyses could not be extended beyond the dorsal midline because of lateral tissue curvature.

### Deletion of Vangl2 in the Grhl3 expression domain causes distal spina bifida

**Fig. 3.**
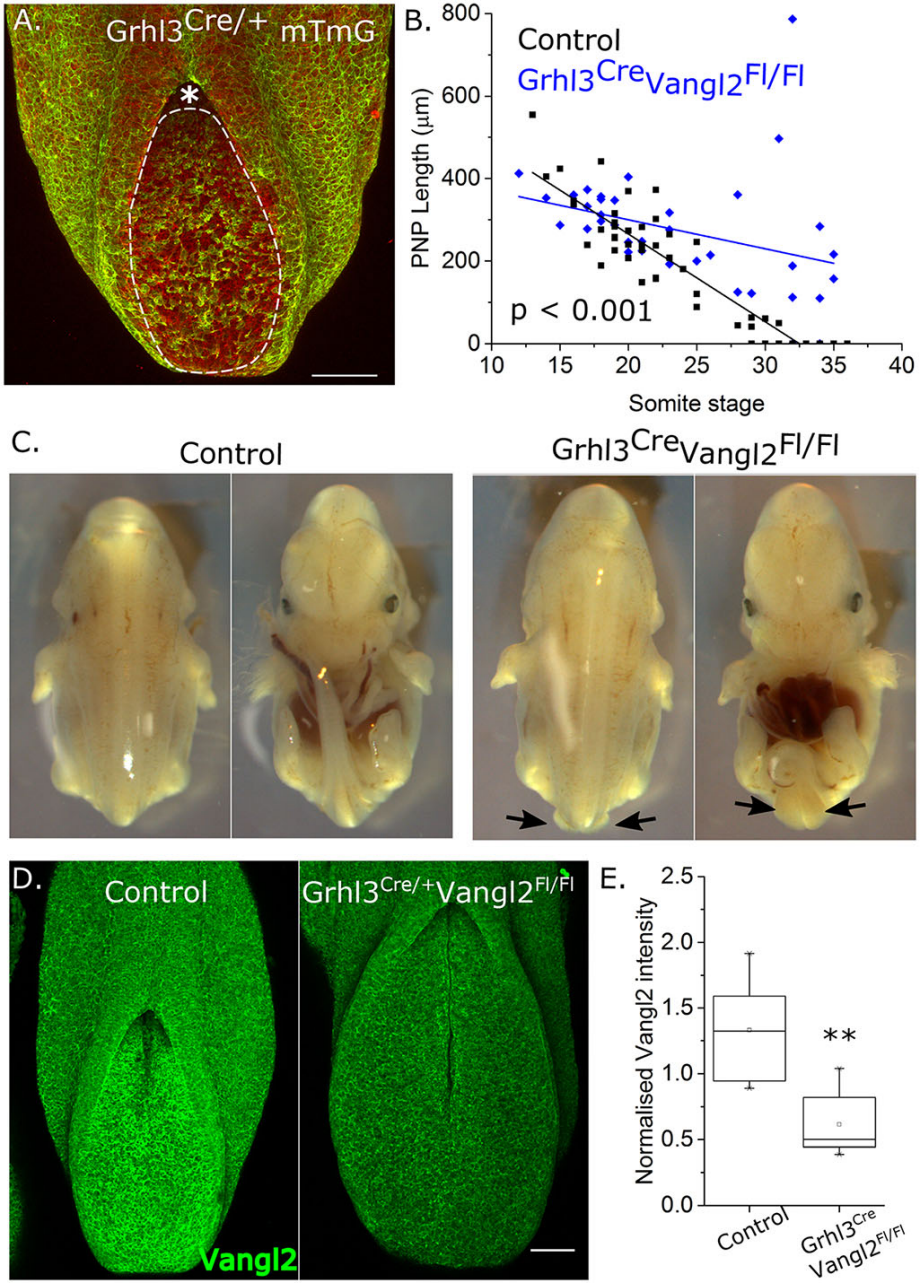
**Vangl2 deletion in the Grhl3 expression domain induces spina bifida.** (A) Representative 16-somite-stage PNP of a *Grhl3^Cre/+^*mTmG embryo, showing Cre-positive lineage tracing (green) in all surface ectoderm cells and numerous neuroepithelial cells (within the dashed boundary) caudal to the zippering point (indicated by an asterisk) at this stage. (B) Quantification of PNP length in control (*Grhl3^+/+^Vangl2^Fl/Fl^* and *Grhl3^Cre/+^Vangl2^Fl/+^*, see Fig. S1B,C) and *Grhl3^Cre/+^Vangl2^Fl/Fl^* E9-E10.5 embryos. Each point represents an individual embryo; *P*<0.001 relates to linear regression slope comparison between genotypes. (C) Representative E14.5 control and *Grhl3^Cre/+^Vangl2^Fl/Fl^* fetuses showing distal spina bifida and curled tail (arrows) in the latter. (D) Representative 18-somite-stage, Vangl2 whole-mount-stained control and *Grhl3^Cre/+^Vangl2^Fl/Fl^* embryo PNPs showing reduced Vangl2 staining intensity in the latter. (E) Quantification of mean neuroepithelial Vangl2 staining intensity normalised to DAPI in control and *Grhl3^Cre/+^Vangl2^Fl/Fl^* embryo PNPs, *n*=6, **P<0.01. Scale bars: 100 µm.

Having identified planar-polarised cell behaviours putatively influenced by PCP signalling, we disrupted this pathway by deleting Vangl2 using Grhl3-driven Cre. *Grhl3^Cre^* recombines in the SE throughout neurulation (Camerer et al., 2010) and also recombines in the PNP particularly at later neurulation stages (Fig. 3A; Fig. S2B). Consistent with *Grhl3^Cre/+^Vangl2^Fl/+^* parent stock mice being morphologically normal, *Grhl3^Cre/+^Vangl2^Fl/+^* embryos did not show any differences in PNP closure compared with *Grhl3^+/+^* embryos (Fig. S2C,D). *Grhl3^+/+^Vangl2^Fl/+^*, *Grhl3*^+/+^ *Vangl2^Fl/Fl^* and *Grhl3^Cre/+^Vangl2^Fl/+^* embryos were therefore considered as ‘controls’.

Vangl2 deletion in the Grhl3 expression domain delayed PNP shortening (Fig. 3B) and narrowing (Fig. S2E) at late-somite stages, without affecting overall embryo elongation (Fig. S2G). This delay in PNP closure tended to become evident around the 25-somite stage (Fig. 3B; Fig. S2E), the approximate somite stage at which Closure 5 forms (Galea et al., 2017). Consistent with this, out of 24 *Grhl3^Cre/+^Vangl2^Fl/Fl^* embryos collected after E10.5, 67% had distal spina bifida (Fig. 3C), 12% had a kinked tail without evident spina bifida and the remaining 21% were morphologically normal. Preceding this, by the 15- to 18-somite stages, Vangl2 expression was abolished in the SE (Fig. S2F,H) and significantly diminished in the neuroepithelium (Fig. 3D,E).

### Vangl2 disruption diminishes surface ectoderm and neuroepithelial cell orientation

**Fig. 4.**
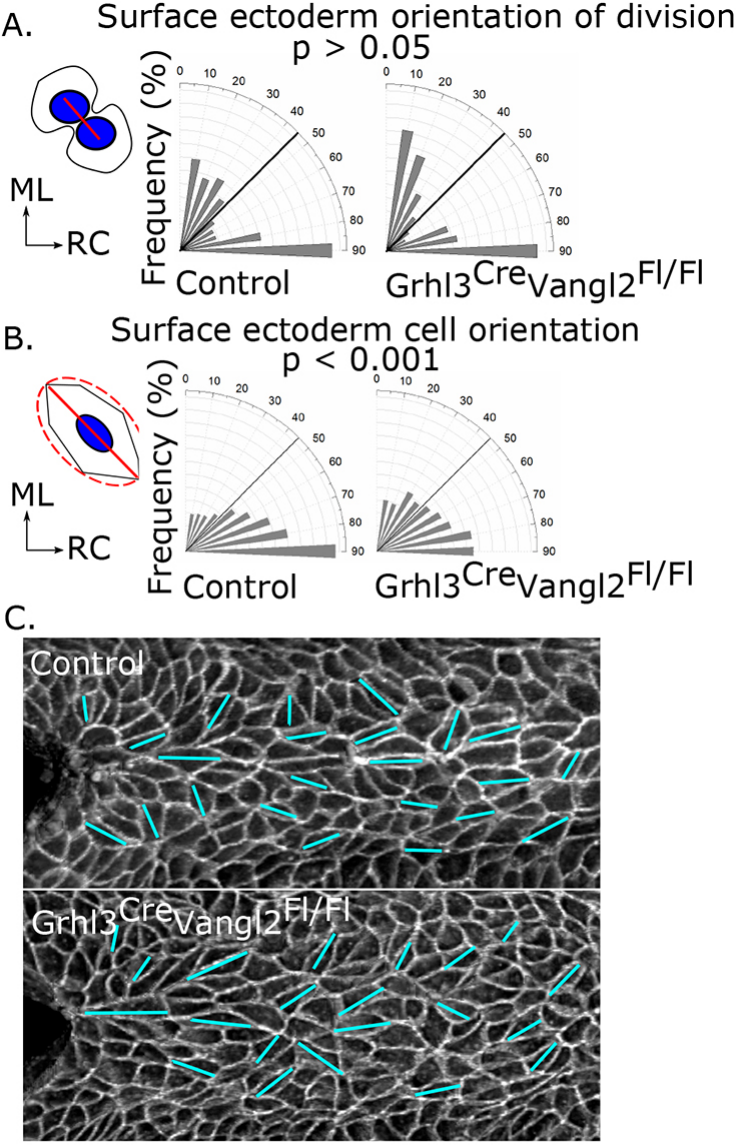
**Vangl2 deletion in the Grhl3 expression domain diminishes polarised orientation of SE cells, but not the orientation of division.** (A) Orientation of SE division in the dorsal midline of 14- to 19-somite stage control (124 divisions from 14 embryos) and *Grhl3^Cre/+^Vangl2^Fl/Fl^* (94 divisions from 13 embryos) embryos. There was no significant difference in SE division orientation between the genotypes. The schematic illustrates the angle of division (red line) of two daughter nuclei (blue circles) of a dividing cell. (B) Orientation of SE cell-long axes along the dorsal midline of control (1080 cells from six embryos) and *Grhl3^Cre/+^Vangl2^Fl/Fl^* (1098 cells from six embryos) embryos. SE cells along the dorsal midline of *Grhl3^Cre/+^Vangl2^Fl/Fl^* embryos were significantly less RC oriented than those in controls. The schematic illustrates the long axis (red solid line) of an ellipse drawn around each cell (red dashed line). (C) Representative surface-subtracted E-cadherin staining of a control and *Grhl3^Cre/+^Vangl2^Fl/Fl^* embryo, showing the orientation of cell-long axes (cyan lines), with the zippering point at the left.

The orientation of SE cell divisions in *Grhl3^Cre/+^Vangl2^Fl/Fl^* embryos was significantly different from random (*P*<0.001 by Chi-square test, compared with the expected proportions in a random situation) and was not significantly different from that in control embryos (Fig. 4A), again showing ML and RC predominance. Vangl2 deletion did not alter the rate of SE division (Fig. S3A). SE cell bodies along the embryonic dorsal midline were also significantly RC oriented in *Grhl3^Cre/+^Vangl2^Fl/Fl^* embryos (*P*<0.001 by Chi-square test), but significantly less so than in control embryos (Fig. 4B,C). Measures of SE cell shape including area, aspect ratio and roundness were not significantly different between genotypes (Fig. S3B-D). Thus, roles of Vangl2 in the SE include the directional polarisation of cell bodies, but not the directional orientation of their division.

**Fig. 5.**
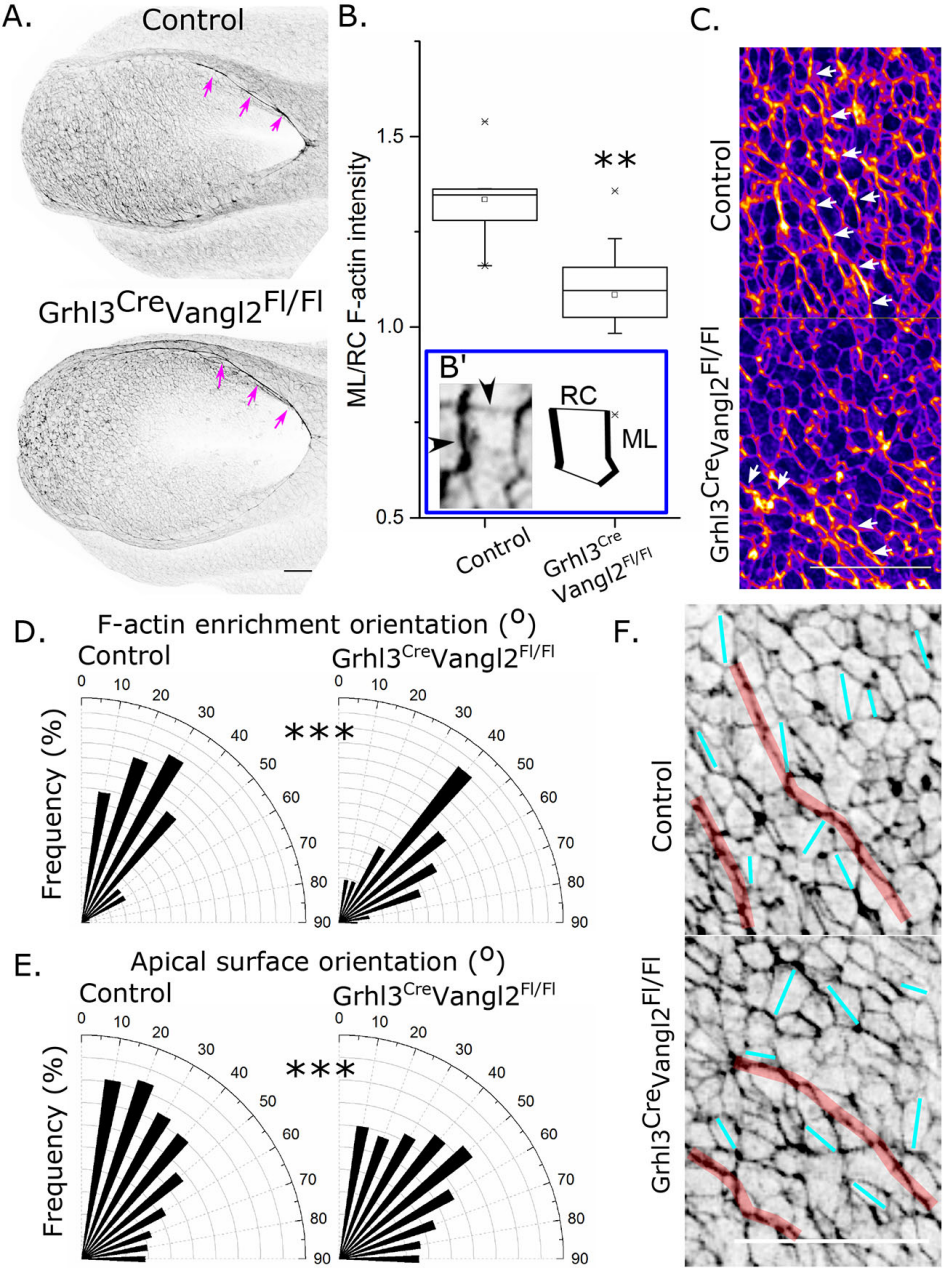
**Vangl2 deletion in the Grhl3 expression domain disrupts the polarised orientation of F-actin and apical cell surfaces in the caudoventral PNP.** (A) Representative phalloidin-stained 16-somite control and *Grhl3^Cre/+^Vangl2^Fl/Fl^* embryos, illustrating the presence of an F-actin cable along the neural folds (magenta arrows; quantified in Fig. S4). (B) The ratio of mediolateral (ML) to rostrocaudal (RC) F-actin staining intensity was quantified in >35 PNP neuroepithelial apical cell surfaces from each of nine control and *Grhl3^Cre/+^Vangl2^Fl/Fl^* 16- to 18-somite embryos to derive an enrichment score (median or ratios per embryo). This process is illustrated in B′ and shown in more detail in Fig. S5A. Enrichment scores were compared between control and *Grhl3^Cre/+^Vangl2^Fl/Fl^* embryos, **P<0.01 versus control. (C) Representative magnified view of phalloidin-stained apical surface of PNP neuroepithelial cells. Fire LUT was used to highlight enrichments (white arrows). (D,E) Analysis of the orientation of supracellular F-actin profiles (D: control, 91 profiles from seven embryos; *Grhl3^Cre/+^Vangl2^Fl/Fl^*, 122 profiles from seven embryos) and apical surface long axis (E: control, 3945 cells from five embryos; *Grhl3^Cre/+^Vangl2^Fl/Fl^*, 4324 cells from five embryos) in control and *Grhl3^Cre/+^Vangl2^Fl/Fl^* embryos. ***P<0.001 comparing proportions in control versus *Grhl3^Cre/+^Vangl2^Fl/Fl^*. (F) Inverted grey LUT of phalloidin-stained apical surface of PNP neuroepithelial cells in a representative control and *Grhl3^Cre/+^Vangl2^Fl/Fl^* embryo, illustrating the orientation of apical surfaces (cyan lines) and F-actin enrichments (red lines). Scale bars: 50 µm.

In the neuroepithelium, we initially investigated whether Vangl2 disruption altered F-actin enrichments. The neural fold F-actin cable and overall F-actin staining intensity were not significantly different between *Grhl3^Cre/+^Vangl2^Fl/Fl^* and control embryos (Fig. 5A; Fig. S4A-C). However, the ML neuroepithelial F-actin profiles were substantially disrupted in *Grhl3^Cre/+^Vangl2^Fl/Fl^* embryos (Fig. 5; Fig. S5). To quantify this, we initially calculated F-actin ML enrichment scores based on the ratio of F-actin staining intensity along ML- versus RC-oriented border, such that ratios greater than 1 indicate ML enriched profiles (illustrated in Fig. S5A). Enrichment scores in control embryos were significantly greater than 1 (*P*<0.001 by one-sample Student's *t*-test), whereas enrichment scores of *Grhl3^Cre/+^Vangl2^Fl/Fl^* embryos were not significantly different from 1 (*P*=0.164) and were significantly smaller than those of control embryos (Fig. 5B,C).

Nonetheless, F-actin profiles were still subjectively visible in the PNP of *Grhl3^Cre/+^Vangl2^Fl/Fl^* embryos (Fig. 5C,F). These supracellular profiles were significantly less ML oriented than in control embryos (Fig. 5C-F). As these profiles form in the apical surface of neuroepithelial cells, we analysed these cells' apical orientations (Fig. S5B). Neuroepithelial apical surfaces were significantly less ML oriented in Vangl2-disrupted embryos (Fig. 5E,F), without substantial differences in their dimensions (Fig. S5C,D). Thus, the roles of Vangl2 in the PNP include ML polarisation of F-actin profiles associated with ML orientation of apical cell surfaces.

### Vangl2 disruption alters caudal neuroepithelial morphology, precluding biomechanical accommodation at late stages of PNP closure

**Fig. 6.**
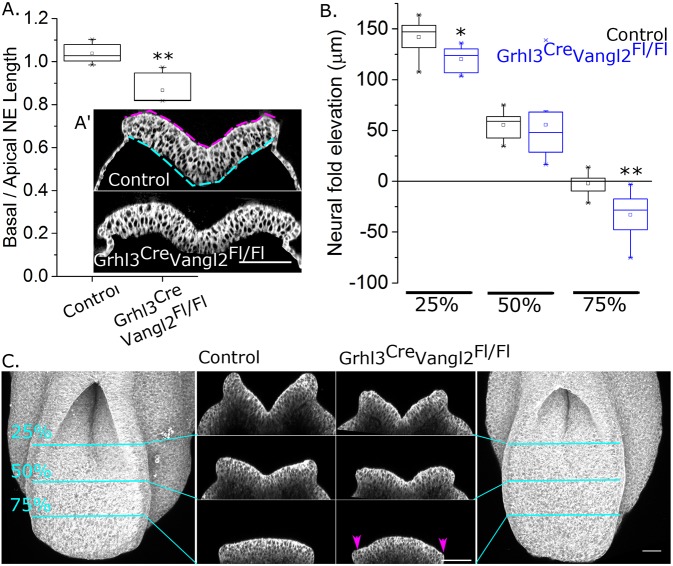
**Vangl2 deletion in the Grhl3 expression domain results in a thinner, more caudally everted PNP neuroepithelium.** (A) Quantification of the mid-PNP ratio between the basal and apical lengths of the neuroepithelium in 16- to 18-somite-stage control and *Grhl3^Cre/+^Vangl2^Fl/Fl^* embryos, *n*=6 per genotype. (A′) Illustrative, digitally enhanced (ImageJ local contrast enhancement and mesoderm removed) PNP optical cross-section through a control and *Grhl3^Cre/+^Vangl2^Fl/Fl^* embryo, showing the ML length of the apical (magenta dashed line) and basal (cyan dashed line) domain of the neuroepithelium in the control. Scale bar: 100 µm. (B) Quantification of neural fold dorsoventral elevation between the apical neuroepithelium ventrally (i.e. median hinge point in the rostral PNP) and the neural fold tips dorsally at 25%, 50% and 75% of the PNP length from the zippering point (indicated in C). Negative values indicate eversion (neural fold tips below apical surface of the PNP), *n*=7 per genotype, 16- to 18-somite stages. (C) Optical cross-sections through representative cell mask-stained control and *Grhl3^Cre/+^Vangl2^Fl/Fl^* embryo PNPs illustrating neural fold elevation or eversion (magenta arrowheads). Scale bars: 50 µm. *P<0.05, **P<0.01 versus control.

Diminished Vangl2 function has previously been reported to alter apical cell intercalation with minimal changes in the epithelial basal domain (Williams et al., 2014). Consistent with a domain-specific effect, the ratio of basal to apical neuroepithelial ML length was significantly smaller in *Grhl3^Cre/+^Vangl2^Fl/Fl^* mid-PNP relative to controls (Fig. 6A). The apical neuroepithelial ML length was not significantly different from the basal length in control embryos (*P*=0.10 by Student's *t*-test paired by embryo) in this minimally elevated region of the neuroepithelium, but the apical length was significantly longer than the basal length in *Grhl3^Cre/+^Vangl2^Fl/Fl^* embryos (*P*=0.01). PNP morphometric analysis also revealed that elevation of the neural folds was significantly diminished in the rostral PNP (25% of the PNP length from the zippering point), but similar at the mid-PNP of *Grhl3^Cre/+^Vangl2^Fl/Fl^* compared with control embryos (Fig. 6B,C). In the caudal PNP (75% of the PNP length from the zippering point), the neural folds were everted in *Grhl3^Cre/+^Vangl2^Fl/Fl^* embryos relative to the apical surface of the neuroepithelium, whereas in control embryos they were flat (Fig. 6B,C).

**Fig. 7.**
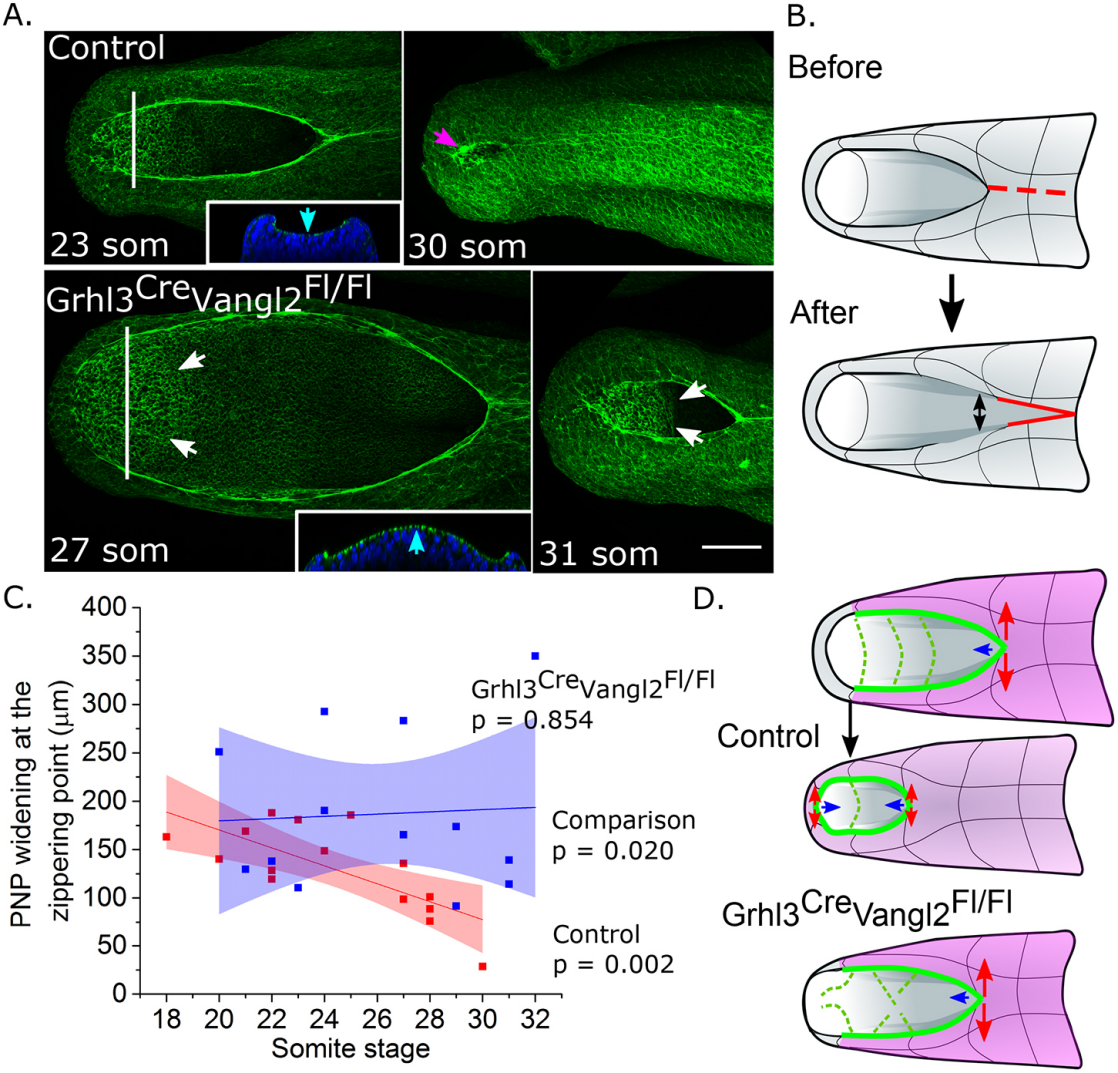
**Vangl2 deletion in the Grhl3 expression domain disrupts biomechanical accommodation normally associated with Closure 5 formation.** (A) Representative phalloidin-stained control and *Grhl3^Cre/+^Vangl2^Fl/Fl^* embryos at the indicated somite stages (som). Control images indicate elevation of the neural folds (cyan arrow in the optical cross-section through the distal PNP at the level of the white line) and subsequent formation of cytoskeletal-rich terminal protrusions characteristic of Closure 5 (magenta arrow at 30 som). *Grhl3^Cre/+^Vangl2^Fl/Fl^* images indicate eversion of the caudal neural plate tissue (cyan arrow in cross-section) resulting in a tissue ‘bulge’ (white arrows) in the region where Closure 5 would usually form (31 som). (B) Schematic illustration of the zippering point laser ablation methodology previously reported (Galea et al., 2017). After laser ablation of the zippering point and recently fused neural tube (dashed red line), the change in PNP width (double-headed arrow) was quantified in live-imaged embryos. (C) Increase in PNP width at the zippering point following laser ablation in control and *Grhl3^Cre/+^Vangl2^Fl/Fl^* embryos at the indicated somite stages. Shaded areas indicate the 95% confidence interval around the linear regression. *P*-values for individual genotypes indicate that the regression slope of the control embryo data is significantly different from 0 and that of the *Grhl3^Cre/+^Vangl2^Fl/Fl^* embryo data is not. The comparison *P*-value indicates a significant overall difference between genotypes based on a linear regression *F*-test. (D) Schematic of the proposed model of PNP closure (in the direction of the blue arrows). In control embryos, the PNP is biomechanically coupled by long-ranging F-actin cables (solid green lines) linking the zippering point to ML-polarised F-actin profiles in the neuroepithelium (dashed green lines), facilitating medial apposition of the neural folds. The zippering point withstands mechanical tension as evidenced by neural fold recoil observed following laser ablation (red arrows). When completion of closure is imminent, this tension is shared with Closure 5, decreasing the magnitude of recoil when the zippering point is ablated. By contrast, disorganised F-actin and cell alignment in the PNP of *Grhl3^Cre/+^Vangl2^Fl/Fl^* embryos is associated with morphologically abnormal Closure 5 and no reduction (biomechanical accommodation) in the magnitude of neural fold recoil with advancing somite stage.

Eversion of the PNP persisted towards the end of spinal neurulation, producing a dorsal ‘bulge’ of midline neuroepithelial tissue at a stage when, in control embryos, the midline neural plate is flat and located between elevating caudal neural folds (Fig. 7A). This tissue bulge intervened between the neural folds of *Grhl3^Cre/+^Vangl2^Fl/Fl^* embryos and prevented the PNP from adopting a Closure 5 morphology: that is, an elliptical PNP encircled by F-actin cables running along elevated neural folds with cytoskeletal-rich protrusions at its caudal canthus (Fig. 7A). The presence of a caudal PNP ‘bulge’ in *Grhl3^Cre/+^Vangl2^Fl/Fl^* embryos was also visible in live-imaged embryos (Fig. S6A). Functions of Closure 5 include facilitation of medial apposition of the neural folds, as demonstrated by laser ablation experiments in which its ablation results in lateral recoil of the neural folds and widening of the caudal PNP (Galea et al., 2017). In keeping with this finding, we observed that the formation of Closure 5 around the 25-somite stage in control embryos was associated with a reduction in PNP widening following laser ablation of the rostral zippering point (Fig. 7C). This suggests that formation of a biomechanically active Closure 5 diminishes or ‘accommodates’ the mechanical stress withstood by the rostral zippering point when completion of closure is imminent. The absence of Closure 5 from *Grhl3^Cre/+^Vangl2^Fl/Fl^* embryos led us to hypothesise that stress accommodation at late developmental stages would not occur in this genotype. Experimental analysis, by laser ablation of the main zippering point in Control and *Grhl3^Cre/+^Vangl2^Fl/Fl^* embryos, strongly supported this hypothesis: there was no reduction in PNP widening following zippering point ablation in *Grhl3^Cre/+^Vangl2^Fl/Fl^* embryos with more than 25 somites, in contrast to controls that exhibited a significant reduction in widening at this stage (Fig. 7C; Fig. S6B,C). We conclude that biomechanical accommodation of mechanical stresses at the zippering point facilitates the final stage of PNP closure under normal circumstances, whereas, in the absence of Vangl2 function, Closure 5 fails to form, the zippering point experiences supranormal mechanical stress and closure fails, leading to distal spina bifida (Fig. 7D).

## DISCUSSION

PCP is crucially required for the initiation of NT closure (Ybot-Gonzalez et al., 2007b). Data from humans as well as mice suggest that it also contributes to completion of spinal closure. Here, we demonstrate that planar-polarised cellular features are evident in both the SE and neuroepithelium of mid- to late-neurulation mouse embryos.

The SE contributes to PNP closure, at least in part, through the formation of zippering protrusions (Rolo et al., 2016) and by secreting signalling molecules (Ybot-Gonzalez et al., 2007a). Planar-polarised divisions in this tissue have previously been reported (Sausedo et al., 1997) and, at later developmental stages when multi-layered skin has formed, core PCP components influence the RC versus dorsoventral predominance of epidermal cell divisions (Oozeer et al., 2017). Established roles of PCP signalling in the skin include the RC orientation of dorsal hair follicles (Devenport and Fuchs, 2008), although whether this phenotype is influenced by the planar-polarised orientation of SE cell bodies earlier in development is not known. Conditional deletion of Vangl1 and Vangl2 with *Cdx2^Cre^* causes distal spina bifida (Chang et al., 2016), similar to that seen with *Grhl3^Cre^* in the present study. *Cdx2^Cre^*-mediated deletion of Vangl1 and Vangl2 resulted in a near-complete (95%) penetrance of distal spina bifida, tail truncation and retarded growth of the hind limbs, whereas *Grhl3^Cre/+^Vangl2^Fl/Fl^* embryos in the present study had a lower penetrance of distal spina bifida and a curled tail similar to that in *Vangl2^Lp/+^* mutants. These differences might be caused by different Cre expression domains [which in the case of *Cdx2^Cre^* is the embryonic ‘posterior, including the epidermis’ (Chang et al., 2016)], or to deletion of Vangl1 as well as Vangl2. In the present study, we found that Vangl2 nonredundantly influenced the orientation of SE cell bodies, but not of planar-polarised cell divisions, suggesting that PCP signalling is active in this cell layer even before NT closure is complete. SE cell morphometric analyses were performed in embryos with 14-19 somites, before PNP dimensions or zippering point tensions diverge significantly between genotypes.

Recombination of *Grhl3^Cre^* is largely SE-specific at early developmental stages, but scattered Cre expression in the neuroepithelium has previously been described (Rolo et al., 2016). Neuroepithelial recombination is most evident from mid-spine neurulation stages, consistent with the expression pattern of the endogenous *Grhl3* gene (Rolo et al., 2016; Gustavsson et al., 2007). In the present study, *Grhl3^Cre^* reduced Vangl2 protein in both the SE and neuroepithelium, although the proportion of recombined neuroepithelial cells was variable, in contrast to the SE, where Cre recombination occurred in effectively 100% of cells. Variability of Cre recombination in the neuroepithelium could account for the incomplete penetrance of the spina bifida phenotype.

Despite this variable reduction in neuroepithelial Vangl2 expression, the detailed morphometric analysis workflows used in this study enabled substantial neuroepithelial tissue- and cell-level disruptions to be documented in *Grhl3^Cre/+^Vangl2^Fl/Fl^* embryos. In chick embryos, PCP-dependent ML contraction of apical F-actin profiles elevates the neural folds by bending the neuroepithelium (Nishimura et al., 2012). To our knowledge, the present study is the first to document such profiles in the mid-neurulation mouse neuroepithelium. We found that Vangl2 disruption prevents the organisation of neuroepithelial apical F-actin into ML-oriented profiles associated with ML orientation of apical cell surfaces*.* By contrast, the biomechanically coupling F-actin cable (Galea et al., 2017) along the neural folds is unaffected. This suggests neurulation-stage epithelia are able to form Vangl2-dependent ML F-actin profiles in the bending neuroepithelium, but use Vangl2-independent mechanisms to form the F-actin cable along the neural folds. Consistent with biomechanical coupling of the PNP being intact, laser ablation of the zippering point caused far-reaching PNP widening in Vangl2-disrupted embryos to a similar extent as in control littermates at somite stages before the formation of Closure 5. The presence and normal appearance of these cables in future spina bifida *Grhl3^Cre/+^Vangl2^Fl/Fl^* embryos suggests they are not, by themselves, sufficient to drive PNP closure.

PCP/Vangl2 signalling is linked to actomyosin contractility through a Dishevelled/Shroom3 pathway involving ROCK proteins in mammalian cells (McGreevy et al., 2015). In E8.5 mouse neural plates, phosphomyosin follows a planar-polarised distribution similar to F-actin and its distribution is disrupted by *Vangl2^Lp/Lp^* mutation (McGreevy et al., 2015). Anisotropic actomyosin distribution suggests that neuroepithelial cells generate mechanical force anisotropically, preferentially in the direction of neural fold apposition. Quantification of the magnitude and distribution of tension within this epithelium will, at the very least, require careful analysis of targeted laser ablations of ML- versus RC-oriented cell borders, which is beyond the scope of the current investigation.

The most marked difference between genotypes observed in this study was eversion of the caudal PNP, which was evident from mid-spine neurulation levels and persisted as a neuroepithelial ‘bulge’ at stages when Closure 5 normally forms. This, together with loss of neuroepithelial F-actin profiles and apical cell surface orientation, suggests that the neuroepithelium is the primary tissue in which loss of Vangl2 precipitates failure of PNP closure. Epithelial bending, as exemplified by NT closure, occurs through a reduction in apical surface length relative to basal length (Nishimura et al., 2012). This was confirmed for control embryos in the present study, but the opposite was seen in the everting neuroepithelium of *Grhl3^Cre/+^Vangl2^Fl/Fl^* embryos. Although the basal neuroepithelium could not be directly investigated in this study of mid- to late neurulation embryos, previous live-imaging analysis of earlier stage embryos (before axial rotation) concluded that extension of basal cellular processes involved in convergent extension was unaffected by homozygous *Lp* mutation of *Vangl2* (Williams et al., 2014).

Apical constriction was also diminished in *Vangl2^Lp/Lp^* embryos prior to Closure 1 (Williams et al., 2014). By contrast, in the present study, apical neuroepithelial surface area in fixed embryos and the normalised overall intensity of F-actin staining were not significantly different between genotypes. It is possible that paraformaldehyde fixation masked dynamic differences in these measures (Goodwin et al., 2016), although distinct supracellular F-actin structures were clearly preserved despite fixation. In live-imaged embryos at mid-spine neurulation stages, the lateral recoil of the neural folds following zippering points laser ablation was not significantly different in *Grhl3^Cre/+^Vangl2^Fl/Fl^* embryos compared with controls. Our previous biomechanical evaluation of PNP stresses demonstrated that the zippering point withstands RC-oriented stress in the direction of body axis elongation as well as ML-oriented stress, which must be overcome to achieve neural fold apposition (Galea et al., 2017). The analyses reported here extend those observations by demonstrating that zippering point tension is dynamic, decreasing at somite stages associated with formation of Closure 5 as a distinct load-bearing structure (Fig. 7D). Laterally tethering stresses are overcome, at least in part, thanks to apical constrictions of the neuroepithelium between the neural folds (Galea et al., 2017). The profiles form part of a larger F-actin network extending to the biomechanically coupling F-actin cable. However, disruption of Closure 5 formation in *Grhl3^Cre/+^Vangl2^Fl/Fl^* embryos owing to eversion and eventual bulging of the caudal PNP prevents stress accommodation (Fig. 7D). Divergence in zippering point stress magnitude between the genotypes, evident from around the 25-somite stage, coincided with the delay in the progression of closure, as indicated by the decrease in PNP length and width. This provides evidence that Closure 5 is not a ‘passive bystander’ in mammalian neurulation, but actively facilitates completion of spinal closure.

A human equivalent of Closure 5 has previously been proposed based on clustering of distal lumbosacral spina bifida lesions (Van Allen et al., 1993; Seller, 1995). The anatomical location of Closure 5 suggests that it might also be involved in the transition to secondary neurulation. Secondary neurulation defects in mice are believed to include the characteristic kinked or looped tail seen in various mutant models (van Straaten and Copp, 2001). This phenotype was also evident in *Grhl3^Cre/+^Vangl2^Fl/Fl^* embryos with spina bifida as well as 38% of embryos which achieved closure. The neuroepithelial dorsoventral eversion observed in the present study, rather than ML widening characteristic of PCP mutants (Curtin et al., 2003; Pryor et al., 2012), suggests that Vangl2 functions extend beyond convergent extension. The cellular basis of convergent extension movements include ML cell intercalation, narrowing tissue dimensions mediolaterally while extending rostrocaudally (Keller et al., 2000). However, it is not clear whether ML cell intercalation movements continue to narrow the late-stage PNP.

In conclusion, the mid- to late-neurulation mouse SE and neuroepithelium show features of planar polarity consistent with their expression of Vangl2. Vangl2 deletion in the *Grhl3^Cre^* domain does not significantly impair the initiation of closure or its progression until approximately the 25-somite stage, but subsequently results in spina bifida. Our findings suggest that the cause of spina bifida in *Grhl3^Cre/+^Vangl2^Fl/Fl^* embryos is failure of Closure 5 formation, resulting in excessive mechanical stress at the rostral zippering point during late stages of closure (Fig. 7D). If applicable to humans, these findings begin to explain why Vangl2 mutations are associated with distal NT lesions in a proportion of human patients (Kibar et al., 2011; Juriloff and Harris, 2012) in whom neurulation has progressed unperturbed along much of the spinal axis, but fails when completion of closure is imminent.

## MATERIALS AND METHODS

### Animal procedures

Studies were performed under the UK Animals (Scientific Procedures) Act 1986 and the Medical Research Council's Responsibility in the Use of Animals for Medical Research (1993). Mice were timed mated overnight and the morning a copulation plug was identified was considered as E0.5. Heterozygous *Grhl3^Cre/+^* and *Vangl2^Fl/Fl^* mice were as previously described (Camerer et al., 2010; Ramsbottom et al., 2014), and were maintained on a C57BL/6 background. To generate parent stock, *Grhl3^Cre/+^* males were mated with *Vangl2^Fl/Fl^* females to generate *Grhl3^Cre/+^Vangl2^Fl/+^* mice, which were morphologically normal. *Grhl3^Cre/+^Vangl2^Fl/+^* males were crossed with *Vangl2^Fl/Fl^* females to generate embryos with the desired genotypes. Yolk sacs were collected from each embryo and genotyping was performed as previously reported (Rolo et al., 2016). Each embryo was assigned a sequential code which did not reflect its genotype, such that all analyses undertaken at developmental stages before a gross phenotype is visible were performed blinded to genotype. No embryos were excluded from analyses. *Gt(ROSA)26Sor^tm4(ACTB-tdTomato,-EGFP)Luo^/J* (mTmG) double fluorescent reporter mice, which show constitutive tdTomato expression, which is converted to EGFP expression in Cre-recombined cells, were as previously described (Muzumdar et al., 2007) and maintained on a homozygous background.

Embryos were harvested at E9-E14.5 as previously described (Pryor et al., 2012) and rinsed in PBS prior to fixation in 4% paraformaldehyde, pH 7.4. E9-E10.5 embryos were imaged using a Leica DC500 camera to measure PNP length and width and overall embryo length in Fiji (Schindelin et al., 2012).

### Whole-mount staining

All images are representative of observations in at least four independent embryos. Rabbit anti-Vangl2 [Millipore clone 2G4, as previously validated (Belotti et al., 2012), 1:100 dilution], mouse anti-E-cadherin (BD Transduction Laboratories clone 36, 1:200 dilution), pMLCII (Cell Signaling Technology 3671T, 1:100 dilution), Alexa Fluor^®^ 568-conjugated phalloidin (Life Technologies) and Depp Red CellMask^®^ were used. Paraformaldehyde-fixed embryos were permeabilised in PBS with 0.1% Triton X-100 (PBT) for 1 h at room temperature, blocked overnight in a 5% BSA/PBT at 4°C and incubated overnight in primary antibody diluted in blocking solution at 4°C. Embryos were then washed three times for 1 h at room temperature in blocking solution, incubated for 2 h at room temperature in a 1:300 dilution of Alexa Fluor^®^ dye-conjugated secondary antibodies (Thermo Fisher Scientific), 1:200 dilution of phalloidin, 1:500 dilution of CellMask and 0.5 µg/ml DAPI in blocking solution. Excess secondary antibody was removed by washing for 1 h in blocking solution and a further two times for 1 h in PBT at room temperature. Images were captured on a Zeiss Examiner LSM880 confocal using a 20×/1.0 NA Plan Apochromat dipping objective. Embryos were typically imaged with X/Y pixel sizes of 0.59 µm and Z-step of 1.0 µm (speed, 8; bidirectional imaging, 1024×1024 pixels). Images were processed with Zen2.3 software and visualised as maximum projections in Fiji. Look-up tables (LUTs) used are in-built in Fiji, including the Fire LUT used to emphasise regions of bright pixels (which appear hot over a darker blue background).

### Morphometric analyses

In order to selectively visualise the most superficial cell layer (surface ectoderm), Z-stacks were digitally resliced such that the dorsoventral axis was aligned to the image *y*-axis and the ML axis along the *x*-axis. An in-house macro was then applied to binarise the image, identify the top (most dorsal) surface and describe a band ∼5 µm deep into the tissue. This band was then used as a mask to subtract everything outside it. The resulting image was then resliced perpendicularly to allow dorsoventral visualisation. The macro and instructions for use are available from the corresponding author on request.

Symmetry around the dorsoventral axis was assumed whenever calculating angles. Orientation of SE division was analysed in surface-subtracted projections rostral to the zippering point. Anaphase/telophase cells were identified based on their characteristic nuclear morphology and an angle was calculated between the centres of the two daughter nuclei. Divisions identified over the closed dorsal NT and more laterally dorsal to the pre-somitic mesoderm were analysed. SE cell morphometric analyses were performed using Tissue Analyser (Aigouy et al., 2016) in Fiji, based on maximum projections of E-cadherin-stained whole mounts. As tissue curvature would substantially alter apparent cell dimensions, only a narrow region approximately five to six cells on either side of the midline over the dorsal NT within one field of view rostral to the zippering point were analysed. Morphometric analysis of the apical surface of the neuroepithelium was performed based on surface-subtracted F-actin whole-mount maximum projections of the relatively flat caudal region of the PNP (∼60-90% of the PNP length from the zippering point). Cells at the margins between the neuroepithelium and SE were not analysed.

PNP neural fold elevation and neuroepithelial cross-section analyses were performed in digitally resliced images of CellMask-stained PNPs. CellMask signal was digitally enhanced postacquisition using sequential local contrast enhancement (CLAHE) in Fiji (block size 50, histogram bins 150, maximum slope 3, then repeated with block size 30, histogram bins 100, maximum slope 3) with 30-pixel rolling ball radius background subtraction before each CLAHE iteration. This equalised signal through the neuroepithelium.

### Laser ablation and live-embryo imaging

Zippering point laser ablations were performed essentially as previously described (Galea et al., 2017). Embryos were dissected from the amnion, positioned in wells cut out of 4% agarose gel in DMEM, submerged in dissection medium and maintained at 37°C throughout imaging. Microsurgical needles from 11-0 Mersilene (TG140-6, Ethicon) and 10-0 Prolene (BV75-3, Ethicon) were used to hold the embryos in place with the PNP pointing upwards while minimising contact with the heart, which continued to beat steadily throughout each experiment. Images were captured on a Zeiss Examiner LSM880 confocal using a 10×/0.5 NA Plan Apochromat dipping objective. Embryos were typically imaged with X/Y pixel sizes of 0.83 µm and Z-step of 3.32 µm, taking ∼2-4 min to image a single PNP using reflection mode (MBS T80/R20 beam filter). Laser ablations were performed using a MaiTai laser (SpectraPhysics Mai Tai eHP DeepSee multiphoton laser, 800 nm wavelength, 100% laser power, 65.94 µs pixel dwell time, 1 iteration). A 300-500 µm line of closed NT roof was ablated along the embryonic midline by ablating each section within the focal plane.

### Statistical analysis

Comparisons between two groups were by Student's *t*-test, accounting for homogeneity of variance in Microsoft Excel or in SPSS (IBM Statistics 22). Comparison of multiple groups was by one-way ANOVA with post hoc Bonferroni in SPSS. Linear regression F-test was in OriginPro 2016 (Origin Labs). Multivariate analysis was by linear mixed models in SPSS, accounting for the fixed effects of genotype and distance from the zippering point in repeated measures from each, with a post hoc Bonferroni. Analysis of PNP widening following laser ablation was performed blind prior to genotyping. Graphs were made in OriginPro and are represented as box plots (whiskers indicate 95% confidence intervals; the box indicates the 25, 50 and 75 percentiles), frequency distributions or scatter plots. Sample sizes are specified in the figure legends and in each case are greater than or equal to *n*=6 control/*Grhl3^Cre/+^Vangl2^Fl/Fl^* embryo pairs from a minimum of four litters based on previous and pilot experiments. *P*<0.05 was considered statistically significant, and all tests were two-tailed.

## Supplementary Material

Supplementary information

## References

[DMM032219C1] Aigouy B., Umetsu D. and Eaton S. (2016). Segmentation and quantitative analysis of epithelial tissues. *Methods Mol. Biol.* 1478, 227-239. 10.1007/978-1-4939-6371-3_13 27730585

[DMM032219C2] Belotti E., Puvirajesinghe T. M., Audebert S., Baudelet E., Camoin L., Pierres M., Lasvaux L., Ferracci G., Montcouquiol M. and Borg J.-P. (2012). Molecular characterisation of endogenous Vangl2/Vangl1 heteromeric protein complexes. *PLoS ONE* 7, e46213 10.1371/journal.pone.0046213 23029439 PMC3460870

[DMM032219C3] Camerer E., Barker A., Duong D. N., Ganesan R., Kataoka H., Cornelissen I., Darragh M. R., Hussain A., Zheng Y.-W., Srinivasan Y. et al. (2010). Local protease signaling contributes to neural tube closure in the mouse embryo. *Dev. Cell* 18, 25-38. 10.1016/j.devcel.2009.11.014 20152175 PMC2822780

[DMM032219C4] Chang H., Smallwood P. M., Williams J. and Nathans J. (2016). The spatio-temporal domains of Frizzled6 action in planar polarity control of hair follicle orientation. *Dev. Biol.* 409, 181-193. 10.1016/j.ydbio.2015.10.027 26517967 PMC5125082

[DMM032219C5] Ciruna B., Jenny A., Lee D., Mlodzik M. and Schier A. F. (2006). Planar cell polarity signalling couples cell division and morphogenesis during neurulation. *Nature* 439, 220-224. 10.1038/nature04375 16407953 PMC1417047

[DMM032219C6] Curtin J. A., Quint E., Tsipouri V., Arkell R. M., Cattanach B., Copp A. J., Henderson D. J., Spurr N., Stanier P., Fisher E. M. et al. (2003). Mutation of Celsr1 disrupts planar polarity of inner ear hair cells and causes severe neural tube defects in the mouse. *Curr. Biol.* 13, 1129-1133. 10.1016/S0960-9822(03)00374-9 12842012

[DMM032219C7] Devenport D. and Fuchs E. (2008). Planar polarization in embryonic epidermis orchestrates global asymmetric morphogenesis of hair follicles. *Nat. Cell Biol.* 10, 1257-1268. 10.1038/ncb1784 18849982 PMC2607065

[DMM032219C8] Escobedo N., Contreras O., Munoz R., Farias M., Carrasco H., Hill C., Tran U., Pryor S. E., Wessely O., Copp A. J. et al. (2013). Syndecan 4 interacts genetically with Vangl2 to regulate neural tube closure and planar cell polarity. *Development* 140, 3008-3017. 10.1242/dev.091173 23760952 PMC3699283

[DMM032219C9] Galea G. L., Cho Y. J., Galea G., Mole M. A., Rolo A., Savery D., Moulding D., Culshaw L. H., Nikolopoulou E., Greene N. D. E. et al. (2017). Biomechanical coupling facilitates spinal neural tube closure in mouse embryos. *Proc. Natl. Acad. Sci. USA* 114, E5177-E5186. 10.1073/pnas.1700934114 28607062 PMC5495245

[DMM032219C10] Goodwin K., Ellis S. J., Lostchuck E., Zulueta-Coarasa T., Fernandez-Gonzalez R. and Tanentzapf G. (2016). Basal cell-extracellular matrix adhesion regulates force transmission during tissue morphogenesis. *Dev. Cell* 39, 611-625. 10.1016/j.devcel.2016.11.003 27923121

[DMM032219C11] Gustavsson P., Greene N. D. E., Lad D., Pauws E., de Castro S. C. P., Stanier P. and Copp A. J. (2007). Increased expression of Grainyhead-like-3 rescues spina bifida in a folate-resistant mouse model. *Hum. Mol. Genet.* 16, 2640-2646. 10.1093/hmg/ddm221 17720888

[DMM032219C12] Juriloff D. M. and Harris M. J. (2012). A consideration of the evidence that genetic defects in planar cell polarity contribute to the etiology of human neural tube defects. *Birth Defects Res. A Clin. Mol. Teratol* 94, 824-840. 10.1002/bdra.23079 23024041

[DMM032219C13] Keller R., Davidson L., Edlund A., Elul T., Ezin M., Shook D. and Skoglund P. (2000). Mechanisms of convergence and extension by cell intercalation. *Philos. Trans. R. Soc. Lond. B Biol. Sci.* 355, 897-922. 10.1098/rstb.2000.0626 11128984 PMC1692795

[DMM032219C14] Kibar Z., Salem S., Bosoi C. M., Pauwels E., de Marco P., Merello E., Bassuk A. G., Capra V. and Gros P. (2011). Contribution of VANGL2 mutations to isolated neural tube defects. *Clin. Genet.* 80, 76-82. 10.1111/j.1399-0004.2010.01515.x 20738329 PMC3000889

[DMM032219C15] Lindqvist M., Horn Z., Bryja V., Schulte G., Papachristou P., Ajima R., Dyberg C., Arenas E., Yamaguchi T. P., Lagercrantz H. et al. (2010). Vang-like protein 2 and Rac1 interact to regulate adherens junctions. *J. Cell Sci.* 123, 472-483. 10.1242/jcs.048074 20067994 PMC2816190

[DMM032219C16] Lu X., Borchers A. G. M., Jolicoeur C., Rayburn H., Baker J. C. and Tessier-Lavigne M. (2004). PTK7/CCK-4 is a novel regulator of planar cell polarity in vertebrates. *Nature* 430, 93-98. 10.1038/nature02677 15229603

[DMM032219C17] Mcgreevy E. M., Vijayraghavan D., Davidson L. A. and Hildebrand J. D. (2015). Shroom3 functions downstream of planar cell polarity to regulate myosin II distribution and cellular organization during neural tube closure. *Biol. Open* 4, 186-196. 10.1242/bio.20149589 25596276 PMC4365487

[DMM032219C18] Merte J., Jensen D., Wright K., Sarsfield S., Wang Y., Schekman R. and Ginty D. D. (2010). Sec24b selectively sorts Vangl2 to regulate planar cell polarity during neural tube closure. *Nat. Cell Biol.* 12, 41-46; sup pp 1-8 10.1038/ncb2002 19966784 PMC2823131

[DMM032219C19] Monier B., Pélissier-Monier A., Brand A. H. and Sanson B. (2010). An actomyosin-based barrier inhibits cell mixing at compartmental boundaries in Drosophila embryos. *Nat. Cell Biol.* 12, 60-69. 10.1038/ncb2005 19966783 PMC4016768

[DMM032219C20] Morris J. K., Rankin J., Draper E. S., Kurinczuk J. J., Springett A., Tucker D., Wellesley D., Wreyford B. and Wald N. J. (2016). Prevention of neural tube defects in the UK: a missed opportunity. *Arch. Dis. Child.* 101, 604-607. 10.1136/archdischild-2015-309226 26681697 PMC4941168

[DMM032219C21] Muzumdar M. D., Tasic B., Miyamichi K., Li L. and Luo L. (2007). A global double-fluorescent Cre reporter mouse. *Genesis* 45, 593-605. 10.1002/dvg.20335 17868096

[DMM032219C22] Nikolopoulou E., Galea G. L., Rolo A., Greene N. D. E. and Copp A. J. (2017). Neural tube closure: cellular, molecular and biomechanical mechanisms. *Development* 144, 552-566. 10.1242/dev.145904 28196803 PMC5325323

[DMM032219C23] Nishimura T., Honda H. and Takeichi M. (2012). Planar cell polarity links axes of spatial dynamics in neural-tube closure. *Cell* 149, 1084-1097. 10.1016/j.cell.2012.04.021 22632972

[DMM032219C24] Oozeer F., Yates L. L., Dean C. and Formstone C. J. (2017). A role for core planar polarity proteins in cell contact-mediated orientation of planar cell division across the mammalian embryonic skin. *Sci. Rep.* 7, 1880 10.1038/s41598-017-01971-2 28500339 PMC5431842

[DMM032219C25] Ossipova O., Chu C.-W., Fillatre J., Brott B. K., Itoh K. and Sokol S. Y. (2015a). The involvement of PCP proteins in radial cell intercalations during Xenopus embryonic development. *Dev. Biol.* 408, 316-327. 10.1016/j.ydbio.2015.06.013 26079437 PMC4810801

[DMM032219C26] Ossipova O., Kim K. and Sokol S. Y. (2015b). Planar polarization of Vangl2 in the vertebrate neural plate is controlled by Wnt and Myosin II signaling. *Biol. Open* 4, 722-730. 10.1242/bio.201511676 25910938 PMC4467192

[DMM032219C27] Prager A., Hagenlocher C., Ott T., Schambony A. and Feistel K. (2017). hmmr mediates anterior neural tube closure and morphogenesis in the frog Xenopus. *Dev. Biol.* 430, 188-201. 10.1016/j.ydbio.2017.07.020 28778799

[DMM032219C28] Pryor S. E., Massa V., Savery D., Greene N. D. E. and Copp A. J. (2012). Convergent extension analysis in mouse whole embryo culture. *Methods Mol. Biol.* 839, 133-146. 10.1007/978-1-61779-510-7_11 22218898 PMC3616368

[DMM032219C29] Ramsbottom S. A., Sharma V., Rhee H. J., Eley L., Phillips H. M., Rigby H. F., Dean C., Chaudhry B. and Henderson D. J. (2014). Vangl2-regulated polarisation of second heart field-derived cells is required for outflow tract lengthening during cardiac development. *PLoS Genet.* 10, e1004871 10.1371/journal.pgen.1004871 25521757 PMC4270488

[DMM032219C30] Rolo A., Savery D., Escuin S., de Castro S. C., Armer H. E. J., Munro P. M. G., Molè M. A., Greene N. D. E. and Copp A. J. (2016). Regulation of cell protrusions by small GTPases during fusion of the neural folds. *eLife* 5, e13273 10.7554/eLife.13273 27114066 PMC4846376

[DMM032219C31] Sausedo R. A., Smith J. L. and Schoenwolf G. C. (1997). Role of nonrandomly oriented cell division in shaping and bending of the neural plate. *J. Comp. Neurol.* 381, 473-488. 10.1002/(SICI)1096-9861(19970519)381:4<473::AID-CNE7>3.0.CO;2-# 9136804

[DMM032219C32] Schindelin J., Arganda-Carreras I., Frise E., Kaynig V., Longair M., Pietzsch T., Preibisch S., Rueden C., Saalfeld S., Schmid B. et al. (2012). Fiji: an open-source platform for biological-image analysis. *Nat. Methods* 9, 676-682. 10.1038/nmeth.2019 22743772 PMC3855844

[DMM032219C33] Seller M. J. (1995). Further evidence for an intermittent pattern of neural tube closure in humans. *J. Med. Genet.* 32, 205-207. 10.1136/jmg.32.3.205 7783170 PMC1050318

[DMM032219C34] van Allen M. I., Kalousek D. K., Chernoff G. F., Juriloff D., Harris M., Mcgillivray B. C., Yong S.-L., Langlois S., Macleod P. M., Chitayat D. et al. (1993). Evidence for multi-site closure of the neural tube in humans. *Am. J. Med. Genet.* 47, 723-743. 10.1002/ajmg.1320470528 8267004

[DMM032219C35] van Straaten H. W. M. and Copp A. J. (2001). Curly tail: a 50-year history of the mouse spina bifida model. *Anat Embryol. (Berl)* 203, 225-237. 10.1007/s004290100169 11396850 PMC4231291

[DMM032219C36] Williams M., Yen W., Lu X. and Sutherland A. (2014). Distinct apical and basolateral mechanisms drive planar cell polarity-dependent convergent extension of the mouse neural plate. *Dev. Cell* 29, 34-46. 10.1016/j.devcel.2014.02.007 24703875 PMC4120093

[DMM032219C37] Ybot-Gonzalez P., Gaston-Massuet C., Girdler G., Klingensmith J., Arkell R., Greene N. D. E. and Copp A. J. (2007a). Neural plate morphogenesis during mouse neurulation is regulated by antagonism of Bmp signalling. *Development* 134, 3203-3211. 10.1242/dev.008177 17693602

[DMM032219C38] Ybot-Gonzalez P., Savery D., Gerrelli D., Signore M., Mitchell C. E., Faux C. H., Greene N. D. E. and Copp A. J. (2007b). Convergent extension, planar-cell-polarity signalling and initiation of mouse neural tube closure. *Development* 134, 789-799. 10.1242/dev.000380 17229766 PMC1839770

