# Supplementary material for: Vangl2 disruption alters the biomechanics of late spinal neurulation leading to spina bifida in mouse embryos
Source: Dis Model Mech. 2018 Mar 1;11(3):dmm032219. doi: 10.1242/dmm.032219 (PMC5897727; doi:10.1242/dmm.032219)
Supplement: Supplementary information [file dmm-11-032219-s1.pdf]

Supplementary Figures

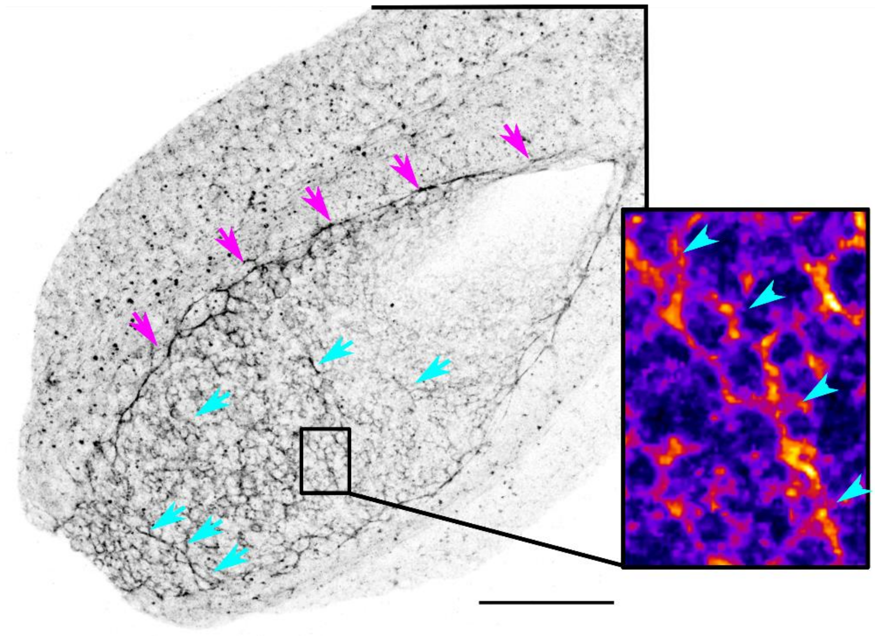

Supplementary Fig. 1: pMLCII staining in the PNP. Wholemount image of PNP stained to show pMLCII. Cyan arrows indicate profile-like arrangements of pMLCII. Magenta arrows indicate pMLCII enrichment in the rostro-caudal cable. Scale = 100 µm.

Disease Models & Mechanisms • Supplementary information

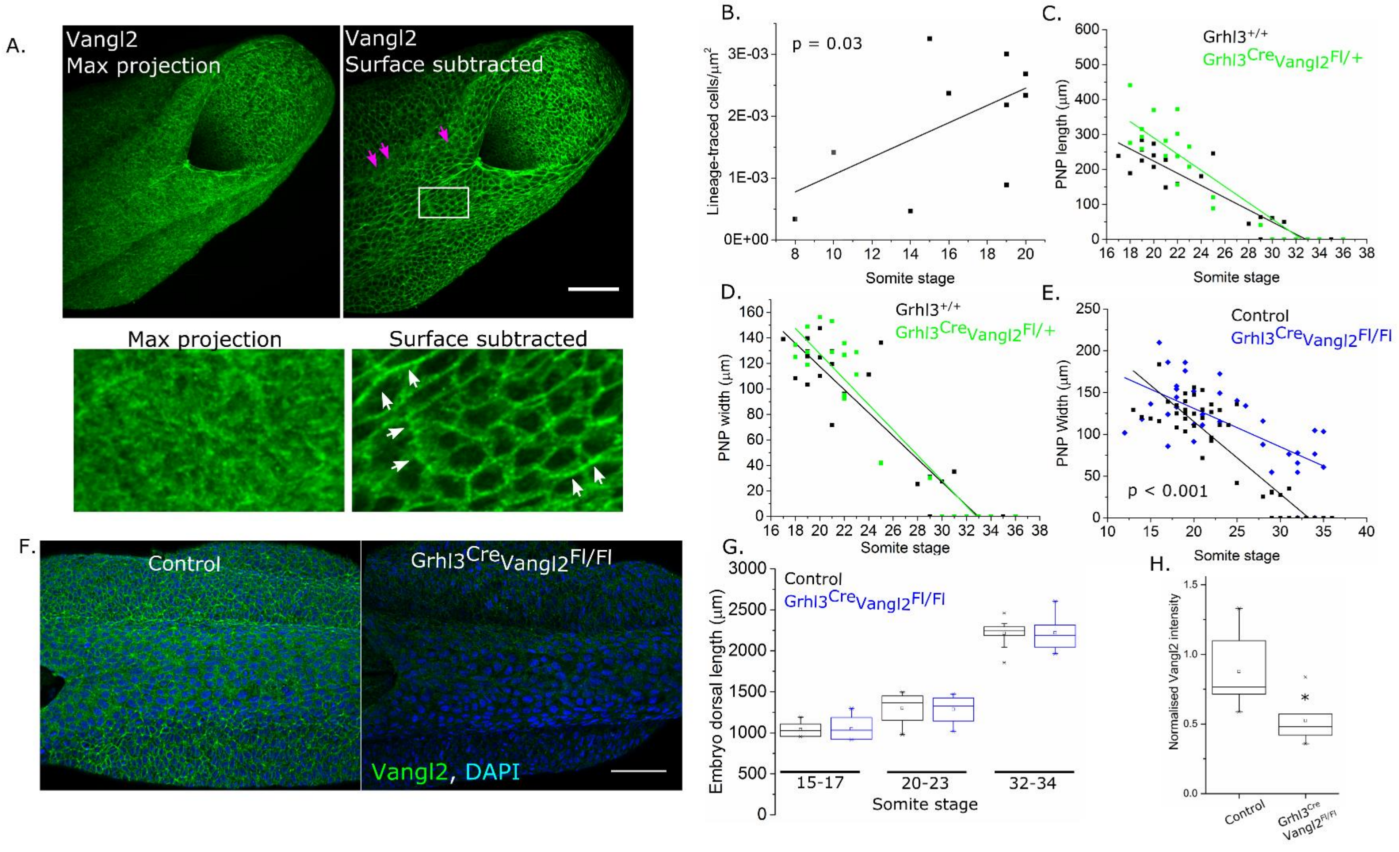

Supplementary Fig. 2: Morphometric measurements of Control and Grhl3$^{Cre/+}$Vangl2$^{Fl/Fl}$ embryos. A) Illustration of the surface subtraction macro used in this study, showing SE cell borders (white arrows in inset box) become visible when fluorescent signal from the underlying closed neural tube is excluded. This macro is less able to separate the SE in regions of marked curvature (magenta arrows showing fuzzy borders as signal from the NT is incompletely excluded). B) Quantification of the number of *Grhl3^{Cre}* lineage traced cells (as shown in Fig. 1a) in the PNP neuroepithelium of embryos at the indicated somite stages. C,D) Quantification of PNP length (C) and width (D) in *Grhl3$^{+/+}$* (with *Vangl2$^{Fl/+}$* or *Vangl2$^{Fl/Fl}$*) versus *Grhl3$^{Cre/+}$Vangl2$^{Fl/+}$* embryo PNPs. The linear regressions are not significantly different from each other. E) Quantification of PNP width in Control and *Grhl3$^{Cre/+}$Vangl2$^{Fl/Fl}$* embryo at the indicated somite stages. F) Representative Vangl2-stained surface subtracted SE from a Control and *Grhl3$^{Cre/+}$Vangl2$^{Fl/Fl}$* embryo, showing marked reduction in Vangl2 staining. Scale bars = 100 µm. G) Quantification of embryo dorsal length (otic vesicle to caudal tip) in Control and *Grhl3$^{Cre/+}$Vangl2$^{Fl/Fl}$* embryos at the indicated somite ranges. H) Quantification of mean Vangl2 staining intensity normalised to DAPI in Control and *Grhl3$^{Cre/+}$Vangl2$^{Fl/Fl}$* embryo SE. * $p < 0.05$, n = 6. H)

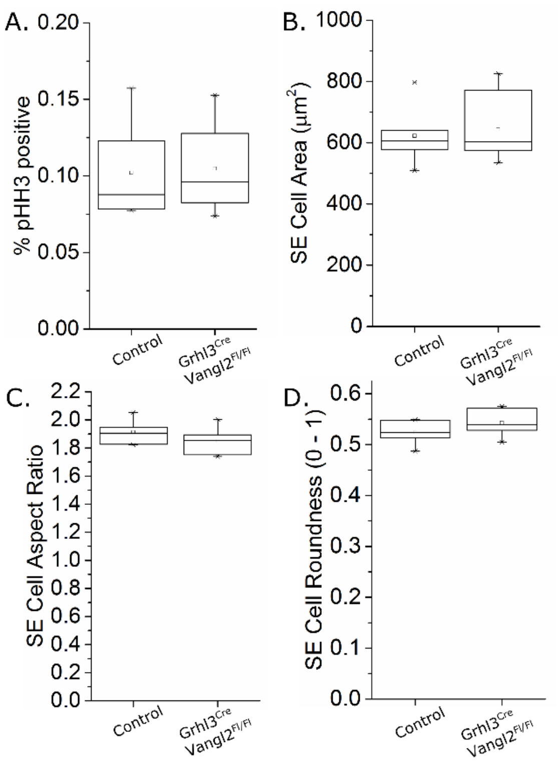

Supplementary Fig. 3: Vangl2 deletion in the Grhl3 expression domain does not alter SE proliferation or cell shape. A) Quantification of the proportion of SE cells stained positive for pHH3 in Control and *Grhl3$^{Cre/+}$Vangl2$^{Fl/Fl}$* embryos, n = 6. B-D) Analysis of SE cell area (B), long to short axis aspect ratio (C) and roundness (D) in Control and *Grhl3$^{Cre/+}$Vangl2$^{Fl/Fl}$* embryos (as in Fig. 3B).

Disease Models & Mechanisms • Supplementary information

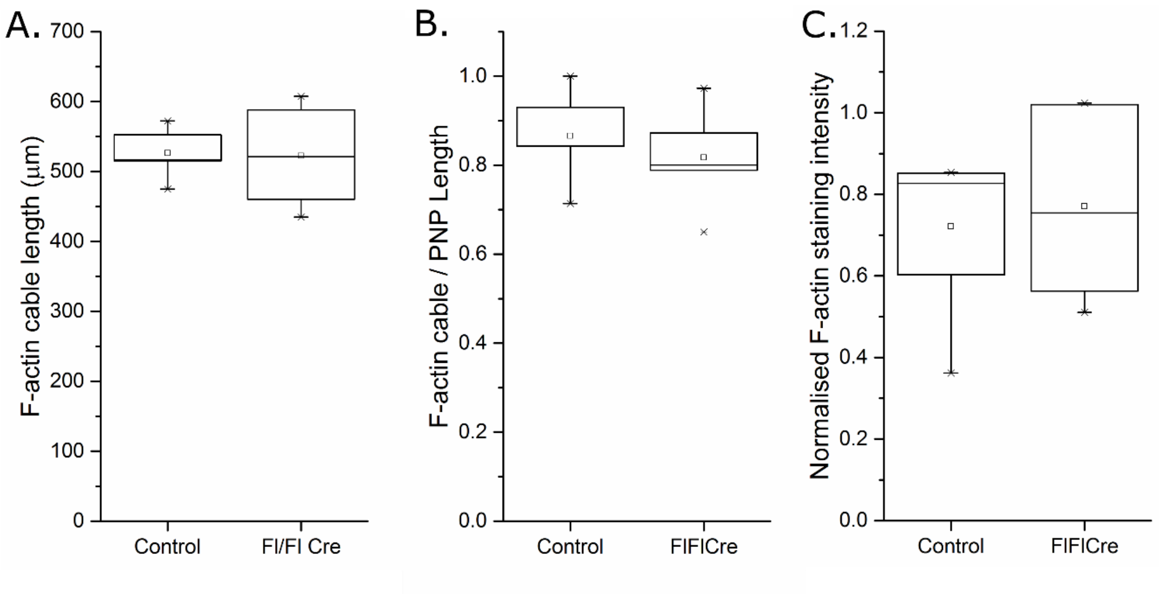

Supplementary Fig. 4: Vangl2 deletion in the Grhl3 expression domain does not grossly disrupt PNP F-actin staining. A,B) Quantification of the absolute length of the F-actin cable along the neural folds (A) and cable length as a proportion of PNP length (B) in 16-18 somite stage Control and *Grhl3*$^{Cre/+}$*Vangl2*$^{Fl/Fl}$ embryos. C) F-actin staining intensity normalised to DAPI staining in Control and *Grhl3*$^{Cre/+}$*Vangl2*$^{Fl/Fl}$ embryos. N = 6 per genotype.

Disease Models & Mechanisms • Supplementary information

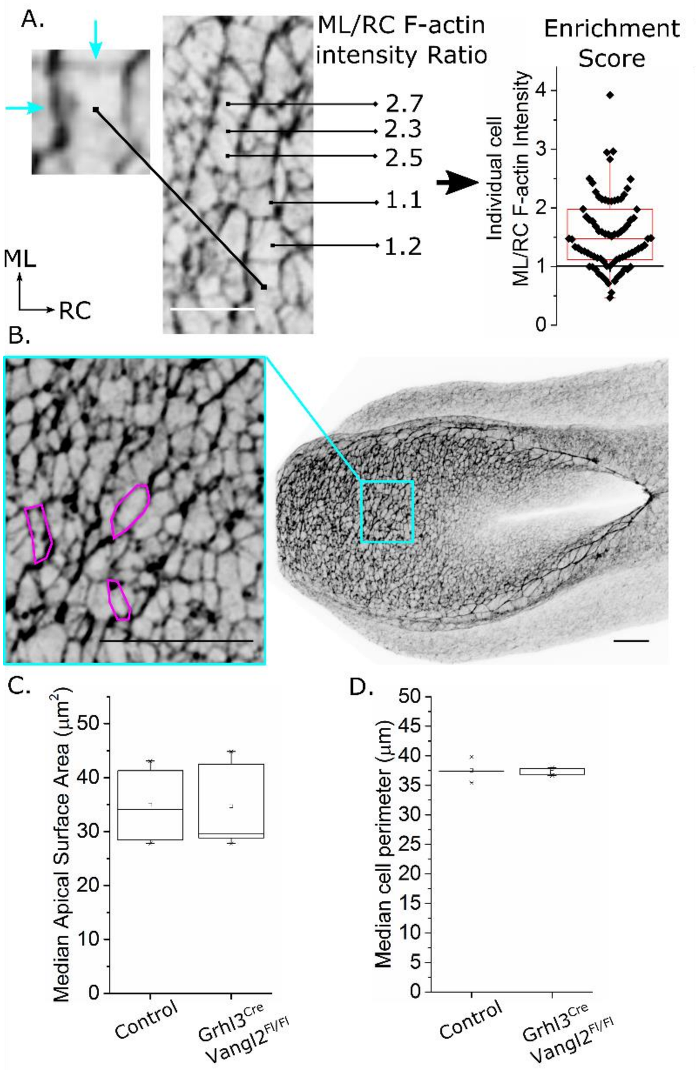

Supplementary Fig. 5: Vangl2 deletion in the Grhl3 expression domain does not alter overall apical dimensions of PNP neuroepithelial cells. A) Illustration of the method used to calculate F-actin enrichment scores. Cells with ML and RC oriented cell borders (cyan arrows) were identified. F-actin staining intensity was calculated along a line drawn over each of these borders, calculating ML/RC F-actin intensity ratios for each cell. The median of these intensity ratios per embryo was calculated as the Enrichment Score. B) Representative 17 somite stage phalloidin stained control embryo illustrating the analysis of PNP apical cell areas (magenta outlines). C,D) Quantification of apical area (C) and perimeter (D) in Control and *Grhl3*$^{Cre/+}$*Vangl2*$^{Fl/Fl}$ embryos. N = 5 per genotype. Scale bars = 50 μm.

Disease Models & Mechanisms • Supplementary information

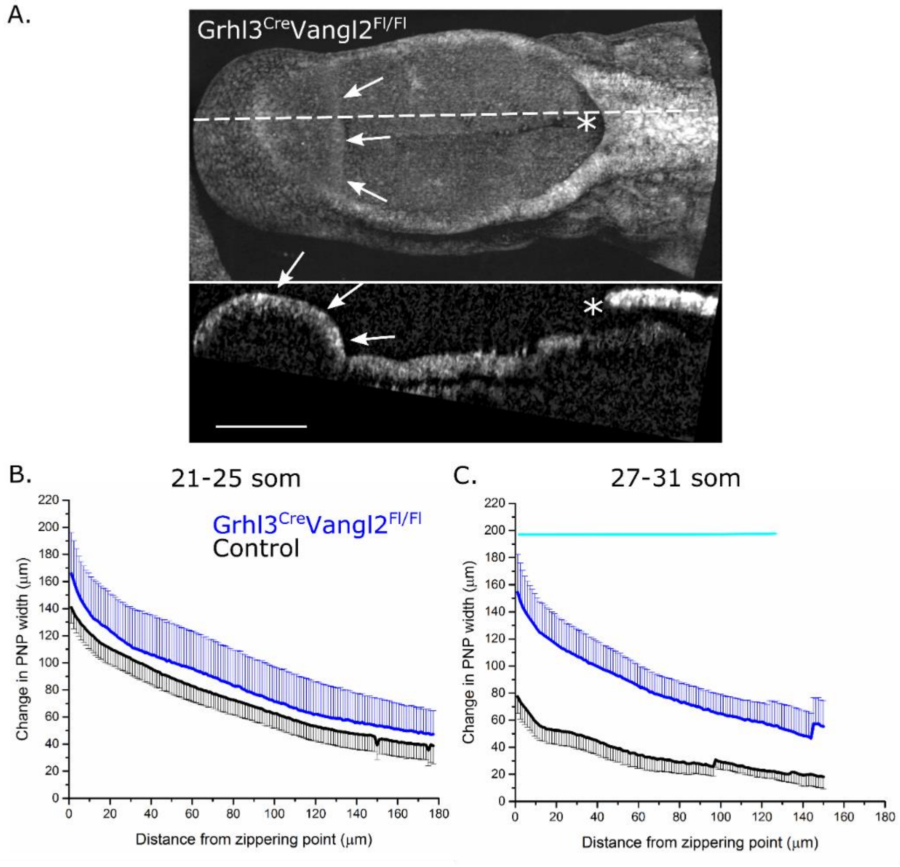

Supplementary Fig. 6: Vangl2 deletion in the Grhl3 expression domain disrupts the biomechanics of PNP closure at late developmental stages. A) Representative live-imaged *Grhl3*$^{Cre/+}$*Vangl2*$^{Fl/Fl}$ embryo showing the caudal PNP "bulge" (arrows) in a dorsoventral view and sagittal cross-section (below) along the dashed line. * = zippering point, scale bar = 200 µm. These images were digitally enhanced in ImageJ (local contrast enhancement) for clarity. B,C) Quantification of the change in PNP width following zippering point ablation in Control and *Grhl3*$^{Cre/+}$*Vangl2*$^{Fl/Fl}$ embryos at 21-25 somites (B: n = 7 and 5, respectively) and 27-31 somite stage (C: n = 6 embryos per genotype). Sequential measurements were taken caudal to the zippering point (at X = 0) in each embryo. The cyan line indicates axial levels that show significantly greater PNP widening in *Grhl3*$^{Cre/+}$*Vangl2*$^{Fl/Fl}$ versus Control embryos by mixed model analysis. Note the marked reduction in PNP widening following ablation (indicative of biomechanical accommodation) between the two somite ranges in Control but not in *Grhl3*$^{Cre/+}$*Vangl2*$^{Fl/Fl}$ embryos.

Disease Models & Mechanisms • Supplementary information
